# *circREEP3* Drives Colorectal Cancer Progression via Activation of FKBP10 Transcription and Restriction of Antitumor Immunity

**DOI:** 10.1002/advs.202105160

**Published:** 2022-03-01

**Authors:** Zhenzhen Chen, Luyun He, Liangbo Zhao, Guangtan Zhang, Zhiwei Wang, Pingping Zhu, Benyu Liu

**Affiliations:** ^1^ School of Life Sciences Zhengzhou University Zhengzhou 450001 China; ^2^ Department of Pathophysiology School of Basic Medical Sciences Zhengzhou University Zhengzhou 450001 China; ^3^ Department of Gastrointestinal Surgery Henan Provincial People's Hospital People's Hospital of Henan University People's Hospital of Zhengzhou University Zhengzhou 450003 China; ^4^ Research Center of Basic Medicine Academy of Medical Sciences Zhengzhou University Zhengzhou 450001 China

**Keywords:** antitumor immunity, *circREEP3*, colorectal cancer, FKBP10

## Abstract

Colorectal cancer (CRC) is one of the most common tumors around the world. Circular RNA is widely involved in tumor progression via unclear mechanisms. Here, *circREEP3* is found to be upregulated in CRC tissues. *circREEP3* upregulation predicts poor patient survival. *circREEP3* knockout suppresses CRC tumorigenesis and metastasis, and impairs stem cell‐like phenotype. Mechanistically, *circREEP3* recruits the chromatin remodeling protein CHD7 to *FKBP10* promoter and activates its transcription. Moreover, *circREEP3* restricts RIG‐1‐dependent antitumor immunity. FKBP10 is highly expressed in CRC tissues and associated with poor prognosis. FKBP10 ectopic expression partially rescues the potential of proliferation and metastasis in *circREEP3*‐deficient CRC cells. Thus, the findings support *circREEP3*‐FKBP10 axis drives CRC progression and may be a critical prognostic marker.

## Graphical abstract

Circular RNA is widely involved in tumor progression via unclear mechanisms. This study shows *circREEP3* upregulation predicts poor patient survival in colorectal cancer (CRC). *circREEP3* promotes CRC tumorigenesis, metastasis, and stem cell‐like phenotype via activating *FKBP10* transcription and restricting RIG‐1‐dependent antitumor immunity. Thus, *circREEP3*‐FKBP10 axis drives CRC progression and may be a critical prognostic marker.

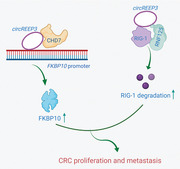

## 1 Introduction

Colorectal cancer (CRC) ranks the third most common cancer in the world and is the second major cause of tumor‐associated death.^[^ ^1^ ^]^ A large proportion of CRC patients are primarily diagnosed with metastasis and nearly most of patients will eventually develop metastatic malignancy.^[^ ^2^ ^]^ The current therapeutic options mainly include surgery, chemotherapy, immunotherapy, and radiation. However, patients with CRC still display a poor five‐year survival rate.^[^ ^2^ ^]^ Nearly 90% of CRC‐induced death is due to distant metastasis and liver is the leading common metastatic site.^[^ ^3^ ^]^ Extensive efforts have been made to explore potential therapeutic targets for CRC intervention. Nevertheless, limited improvement has been made to increase the five‐year survival rate. Therefore, more investigations are critical to fully understand the molecular mechanism underlying CRC pathogenesis and metastasis.

Circular RNAs (circRNAs) are a new member of noncoding RNAs generated through back‐splicing and characterized by a covalent bond linking their 3′ and 5′ end.^[^ ^4^ ^]^ CircRNAs were primarily discovered over 45 years ago and considered as splicing byproducts.^[^ ^5^, ^6^ ^]^ CircRNAs can originate from exons, introns, or exon‐introns.^[^ ^7^ ^]^ Recent reports have indicated that circRNAs are abundantly expressed in diverse tissues in a cell‐type‐specific manner.^[^ ^8^ ^]^ Most circRNAs are highly conserved across species.^[^ ^9^ ^]^ Emerging studies have uncovered that circRNAs exert critical functions in various biological processes by acting as microRNA (miRNA) sponges, transcription regulators, protein partners, and so on.^[^ ^10^ ^]^ For instance, mitochondria‐located circRNA SCAR regulates cirrhosis caused by high fat diet.^[^ ^11^ ^]^ circRNA Cdr1as is critical for brain function by acting as miR‐7 and miR‐671 sponges.^[^ ^12^ ^]^ circRNAs are also widely implicated in the regulation of immunity and tumorigenesis.^[^ ^13^, ^14^ ^]^ We previously found that circKcnt2 and circZbtb20 are essential modulators in group III innate lymphoid cell (ILC3) homeostasis and colitis.^[^ ^15^, ^16^ ^]^ We have also identified cis‐HOX drives colorectal tumor‐initiating cell (TIC) self‐renewal.^[^ ^17^ ^]^ However, how circRNAs regulate CRC initiation and metastasis still remains poorly understood.

FK506‐binding protein 10 (FKBP10), a member of the FKBP subfamily immunophilins, is a chaperone and directly interacts with collagen I.^[^ ^18^ ^]^ FKBP10 mutation causes collagen‐related illnesses such as osteogenesis imperfecta by decreasing collagen secretion.^[^ ^19^, ^20^ ^]^ FKBP10 is identified to be a potential drug target for idiopathic pulmonary fibrosis (IPF).^[^ ^20^ ^]^ Recent works have also emphasized an emerging oncogenic role of FKBP10.^[^ ^21^, ^22^ ^]^ For example, FKBP10 overexpression promoted lung cancer growth.^[^ ^21^, ^22^ ^]^ FKBP10 downregulation diminished renal cell carcinoma propagation.^[^ ^23^ ^]^ Nevertheless, its functions in CRC progression are largely unknown.

Here, we show that circRNA *circREEP3* (originating from *REEP3* gene transcript; circRNA symbol, hsa_circRNA_400564) is upregulated in invasive CRC cells. *circREEP3* promotes CRC proliferation and metastasis. In mechanism, *circREEP3* recruits the chromatin remodeler CHD7 to initiate FKBP10 transcription in the nucleus and inhibits anti‐tumor immunity by enhancing RNF125‐dependent degradation of RIG‐1 in the cytoplasm. Our findings indicate that targeting *circREEP3* may prevent CRC progression, adding an additional layer for circRNA functions and CRC regulations.

## 2 Results

### 2.1 *circREEP3* Is Highly Expressed in CRC

**Figure 1:**
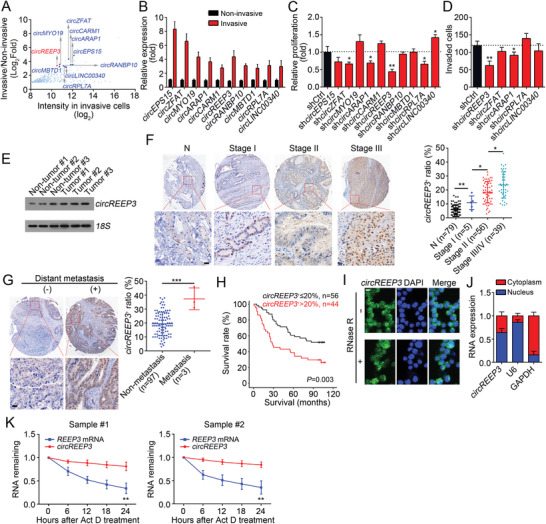
*circREEP3* is highly expressed in CRC. A) The most differentially expressed circRNAs in invasive CRC cells compared to those in noninvasive tumor cells were presented. Transwell assay was performed using CRC sample cells. The cells in the lower chamber were considered as invasive tumor cells while the cells in the upper chamber were considered as noninvasive cells. B) The ten most highly expressed circRNAs in invasive CRC cells were selected and their expression levels were validated through qRT‐PCR. C) CCK8 assay was performed to evaluate cell proliferation ability after circRNA knockdown in CRC sample cells. D) Transwell assay was carried out to examine invasion potential after circRNA silencing in CRC tumor cells. E) *circREEP3* expression in paired CRC tumor tissues and adjacent normal tissues was measured by Northern blotting. 18S acted as the loading control. F) *circREEP3* levels were analyzed in CRC tissue array containing 79 peri‐tumor, 5 stage I, 56 stage II, 39 stage III/IV tissues by in situ hybridization. Typical images containing global views and magnified views were presented in the left panel. Ratios of circRNA positive cells in each sample was calculated and shown in the right panel. Scale bar, 100 µm. G) *circREEP3* levels in CRC tissues with distant metastasis or not were measured by in situ hybridization. H) Survival rate was analyzed by Kaplan–Meier method. Samples were divided into *circREEP3* high (*circREEP* ^+^ ≤ 20%) and low (*circREEP* ^+^ > 20%) subgroups based on *circREEP3* median value (20%). I) Fluorescence in situ hybridization was used to test *circREEP3* expression in RNase‐R treated CRC sample cells. Scale bar, 10 µm. J) Subcellular location of circRNA in CRC sample cells was analyzed by qPCR. K) RNA abundance was evaluated after treatment with Actinomycin D (Act D, 2 µg mL^−1^). *P < 0.05, **P < 0.01, and ***P < 0.001. Data were analyzed by an unpaired Student's *t*‐test and shown as means ± SD. Data are representative of at least three independent experiments.

For CRC patients, metastasis is the leading cause of mortality. We sought to explore how circRNAs regulate CRC metastasis. CRC sample cells were cultured and used for transwell assay. The invasive and noninvasive sample cells were collected for circRNA microarray analysis. According to the expression levels in invasive cells and fold change, ten circRNAs (conserved between mouse and human)^[^ ^24^ ^]^ were selected and presented (**Figure** **1**A). Their upregulation was validated by quantitative real‐time PCR (qRT‐PCR) (Figure 1B). Besides, their identities as circRNAs were verified by Sanger sequencing, PCR (Figure S1A,B, Supporting Information) and RNase R digestion (Figure S1C, Supporting Information). To screen functional circRNAs, we knocked them down and confirmed the efficiency (Figure S1D, Supporting Information). Of note, circRNA depletion did not affect the expression levels of their cognate linear transcripts (Figure S1E, Supporting Information). Then CCK8 assay was performed and five circRNAs (*circZFAT*, *circARAP1*, *circREEP3*, *circRPL7A*, and *circLINC00340*) were found to regulate tumor cell proliferation (Figure 1C). Transwell assay was further conducted to evaluate the effect of these five circRNAs on metastasis. We found that *circREEP3* knockdown significantly inhibited CRC invasion (Figure 1D). Thus, *circREEP3* was selected for further investigation.

*circREEP3* is formed by back‐splicing of *REEP3* transcripts from exon 2 to exon 5 (Figure S1F, Supporting Information). Northern blot also showed that *circREEP3* was upregulated in CRC tissues compared to adjacent nontumor tissues (Figure 1E). A tumor tissue microarray indicated that *circREEP3* level was increased in CRC tissues and positively correlated with clinical severity, metastasis and prognosis (Figure 1F–H and Table S1, Supporting Information). Through fluorescence in situ hybridization (FISH) using *circREEP3* specific probes, we found that *circREEP3* was expressed in the cytoplasm and nucleus (Figure 1I), which was validated by nuclear and cytoplasmic fractionation assay (Figure 1J). We also observed that *circREEP3* was more stable than *REEP3* mRNA after Actinomycin D treatment (Figure 1K), further supporting it as a circRNA.

### 2.2 *circREEP3* Promotes CRC Growth and Metastasis

**Figure 2:**
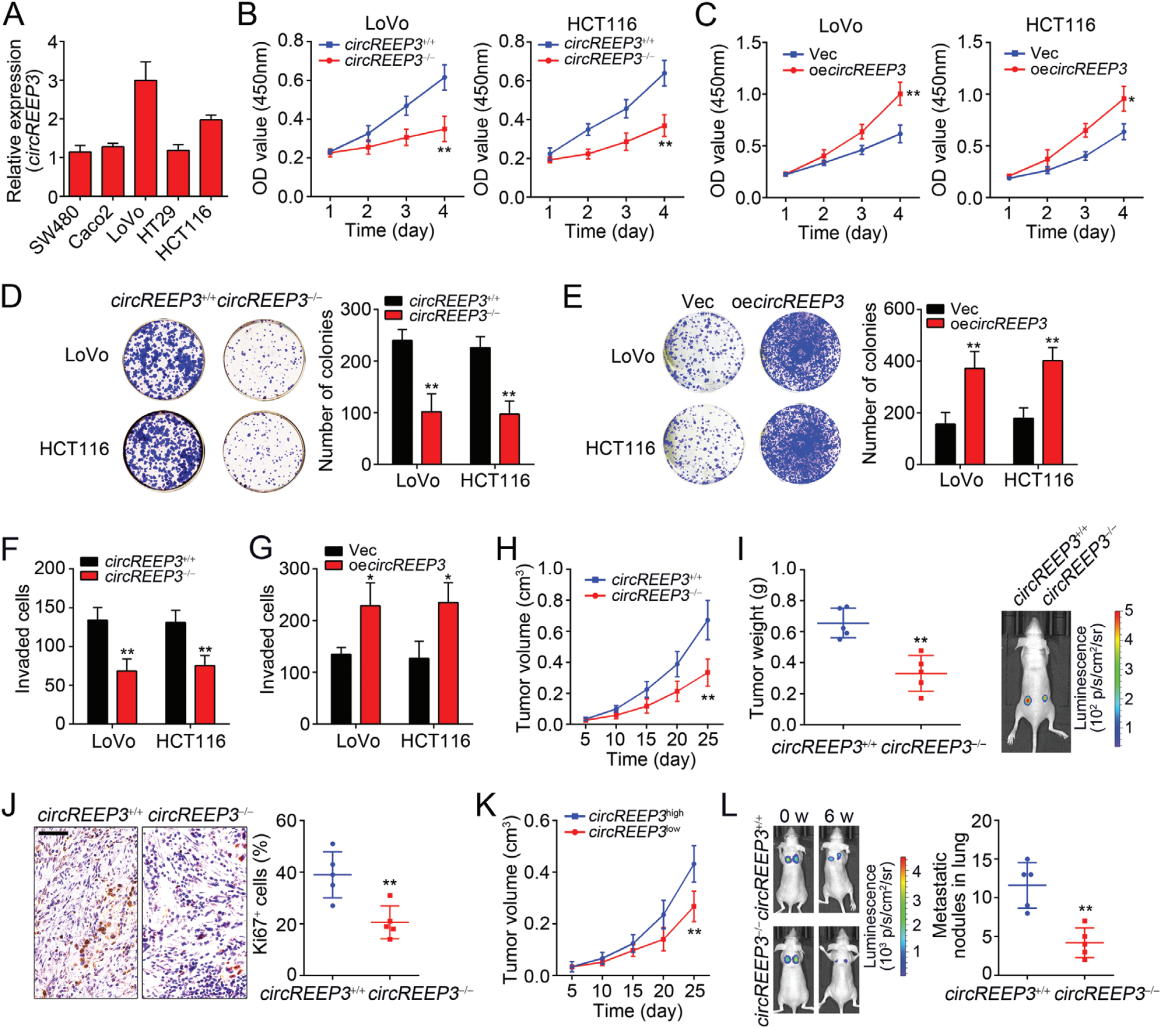
*circREEP3* promotes CRC growth and metastasis. A) Relative expression of *circREEP3* in CRC cell lines was measured by qPCR. B) CCK8 assay for analysis of cell proliferation ability in *circREEP3* ^+/+^ or *circREEP3^−^* ^/−^ LoVo and HCT116 cells. C) CCK8 assay for analysis of cell proliferation ability in *circREEP3*‐overexpressing (oe) or control LoVo and HCT116 cells. Vec, empty vector. D) Colony formation assay was conducted to test cell proliferation using *circREEP3* ^+/+^ or *circREEP3* ^−/−^ LoVo and HCT116 cells. E) Colony formation assay was conducted to test cell proliferation using *circREEP3*‐overexpressing or control LoVo and HCT116 cells. F) Transwell assay using *circREEP3* ^+/+^ or *circREEP3* ^−/−^ LoVo and HCT116 cells was carried out. Invaded cells were calculated. G) Transwell assay using *circREEP3*‐overexpressing or control LoVo and HCT116 cells was carried out. H) *circREEP3* ^+/+^ or *circREEP3* ^−/−^ LoVo cells were used for xenograft assay. Tumor volumes were measured every 5 d. *n* = 5 for each group. I) Tumor weights were determined on day 25 postinjection. Typical luciferase images of *circREEP3* ^+/+^ or *circREEP3* ^−/−^ tumors were in the right. *n* = 5 for each group. J) Immunohistochemistry (IHC) analysis for Ki67 expression in tumor tissues of (I). Percentage of Ki67^+^ cells was calculated and shown in the right panel. Scale bar, 100 µm. K) *circREEP3* highly (*circREEP3* ^high^) or lowly (*circREEP3* ^low^) expressed CRC tumor cells were used for xenograft assay through NSG (NOD/Scid/Il2rg) mice. Tumor volumes were calculated every 5 d. L) Potential of lung metastasis was measured through vein tail injection of *circREEP3* ^+/+^ or *circREEP3* ^−/−^ LoVo cells. Representative images were shown in the left panel. The number of metastatic nodules in the lung was calculated and presented in the right panel. *n* = 5 mice each group. w, week. *P < 0.05 and **P < 0.01. Data were analyzed by an unpaired Student's *t*‐test and shown as means ± SD. Data are representative of at least three independent experiments.

We then analyzed *circREEP3* expression in CRC cell lines and fount that it showed the highest levels in LoVo and HCT116 cells (**Figure** **2**A). To further determine the physiological roles of *circREEP3*, we sought to generate *circREEP3*‐deficient LoVo and HCT116 cells. circRNA formation is dependent on the flanking complementary elements.^[^ ^7^ ^]^ We screened out the complementary sequences in the introns flanking *circREEP3* and validated its necessity for *circREEP3* formation through minigene assay (Figure S2A, Supporting Information). We then deleted the downstream complementary element of genome to generate *circREEP3*‐deficient LoVo and HCT116 cells via CRISPR/Cas9 technology (Figure S2B, Supporting Information). *circREEP3* deletion was validated by PCR and northern blot (Figure S2C,D, Supporting Information). Of note, *circREEP3* deletion did not affect the expression of its maternal gene REEP3 (Figure S2E,F, Supporting Information).

We first tested the effects of *circREEP3* deletion on CRC growth. We found that *circREEP3* knockout significantly suppressed the growth of LoVo and HCT116 cells (Figure 2B). On the contrary, *circREEP3* overexpression increased their proliferation ability (Figure 2C and Figure S2G, Supporting Information). Colony formation assay further supported this finding (Figure 2D,E). Then transwell assay was carried out and we found that *circREEP3* deficiency impaired CRC invasion and vice versa (Figure 2F,G). The role of *circREEP3* knockout on tumor growth was then evaluated. Luciferase‐tagged *circREEP3*‐deficient LoVo cells were subcutaneously injected into the flanks of BALB/c nude mice. Tumor volumes were measured every 5 d and tumor sizes were determined 25 d after injection. We found that *circREEP3* knockout significantly decreased tumor volumes and sizes (Figure 2H,I). The Ki67 positive cells of the xenograft tumor tissues derived from *circREEP3*‐deficient LoVo cells were fewer than that from wild‐type LoVo cells (Figure 2J). We then performed patient‐derived xenografts. We isolated *circREEP3*‐high (*circREEP3* expression levels were higher than the average levels) or *circREEP3*‐low (*circREEP3* expression levels were lower than the average levels) expression CRC sample cells with qPCR and injected them into NSG (NOD/Scid/Il2rg) mice. *circREEP3* low expression sample cells grew slower (Figure 2K). Next, the in vivo effect of *circREEP3* toward metastasis was examined. We found that *circREEP3* knockout remarkably inhibited tumor metastasis in lung (Figure 2L). Above data suggest that *circREEP3* promotes CRC progression and metastasis.

### 2.3 *circREEP3* Contributes to CRC Stem Cell Phenotype

**Figure 3:**
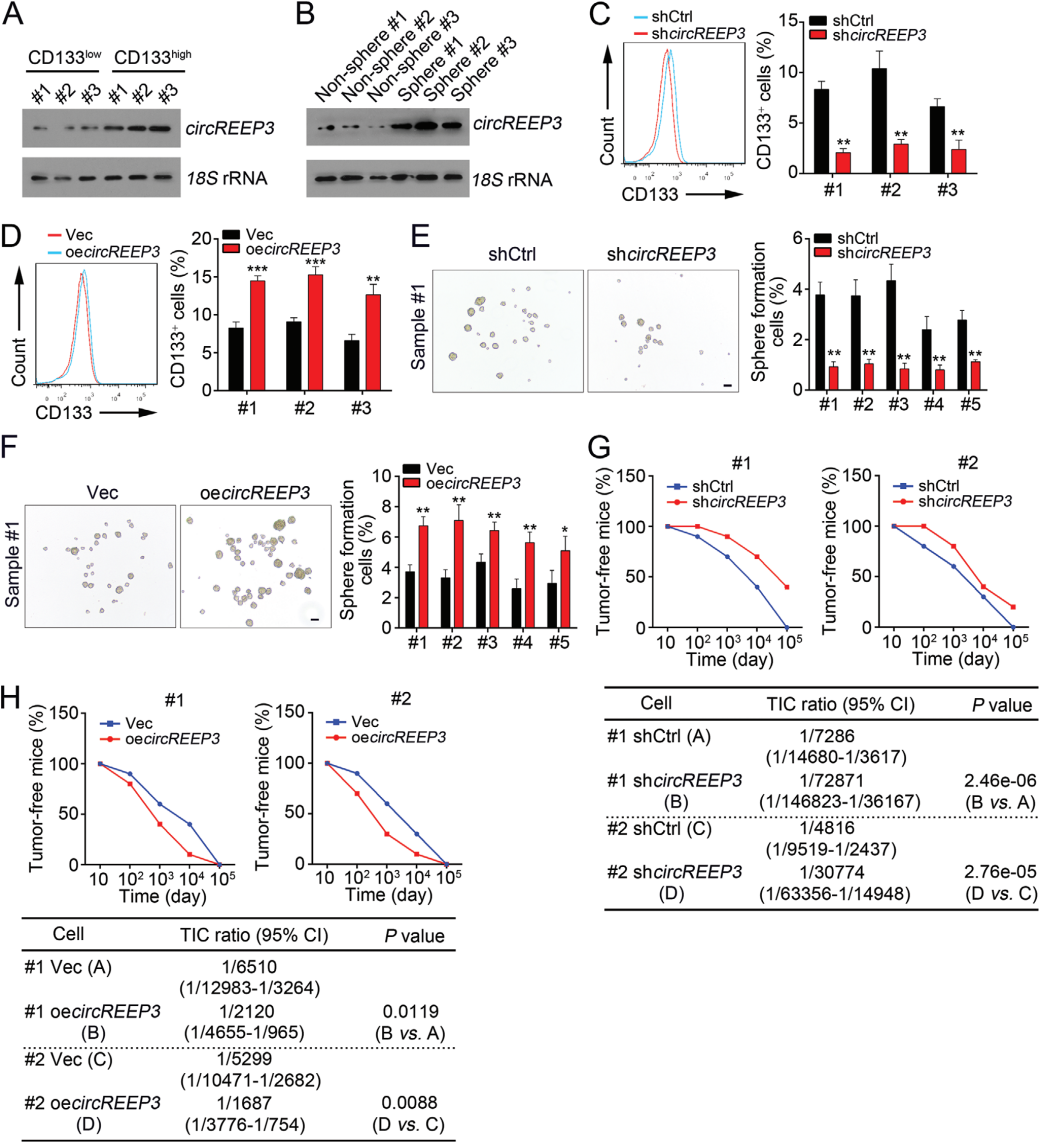
*circREEP3* contributes to CRC stem cell phenotype. A) CRC sample cells were sorted by gating on CD133 and divided into CD133 high (CD133^high^) and low (CD133^low^) subgroups. Then Northern blotting was performed to detect *circREEP3* expression. 18S was the loading control. B) CRC sample cells were used for sphere formation. Then *circREEP3* levels were measured by Northern blotting. 18S was the loading control. C) CRC sample cells with *circREEP3* silencing were used for sphere formation, followed by detection of CD133 expression through FACS analysis. shCtrl, control shRNA. D) *circREEP3* knockdown and control CRC sample cells were used for sphere formation, followed by detection of CD133 expression through FACS analysis. Vec, empty vector. E,F) Sphere formation assay using *circREEP3*‐silenced or overexpressing CRC cells. G,H) Tumor initiation assay using *circREEP3* silenced or overexpressing CRC sample cells were conducted and the ratios of tumor‐free mice were calculated. *n* = 10 for each group. *P < 0.05 and **P < 0.01. Data were analyzed by an unpaired Student's *t*‐test and shown as means ± SD. Data are representative of at least three independent experiments.

CRC stem cells are characterized by powerful potential of self‐renewal and differentiation.^[^ ^25^ ^]^ These cells defined by CD133 expression play vital roles in tumor growth, metastasis, and recurrence.^[^ ^25^, ^26^ ^]^ We noticed that *circREEP3* expression was higher in CD133^high^ CRC sample cells, in which CD133^+^ cells were higher than the average ratios of CD133^+^ cells in all samples (**Figure** **3**A). Besides, *circREEP3* level was lower in nonsphere cells (Figure 3B), suggesting *circREEP3* may regulate stemness. *circREEP3* knockdown also reduced the ratio of CD133^+^ cells and vice versa (Figure 3C,D). Moreover, *circREEP3* silencing inhibited the sphere formation and tumor initiation capacities, and vice versa (Figure 3E–H).

### 2.4 *circREEP3* Initiates FKBP10 Transcription

**Figure 4:**
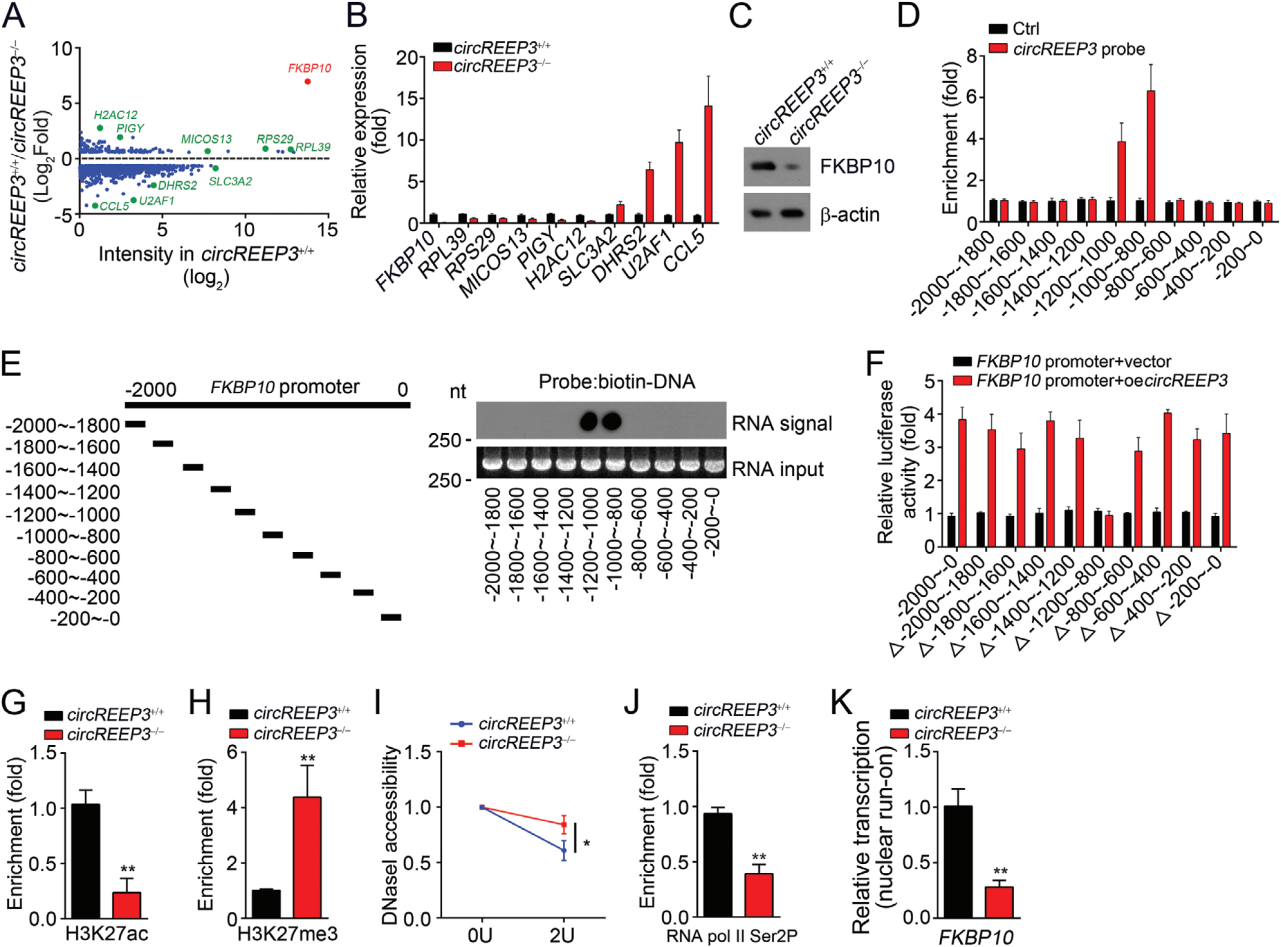
*circREEP3* initiates FKBP10 transcription. A) Differentially expressed genes in *circREEP3* ^+/+^ or *circREEP3* ^−/−^ LoVo cells. B) qPCR validation of the most downregulated or upregulated genes in *circREEP3* ^−/−^ LoVo cells. C) Western blotting analysis for FKBP10 expression in *circREEP3* ^−/−^ LoVo cells. D) ChIRP (Chromatin Isolation by RNA Purification) assay was performed using biotin‐labeled *circREEP3* probes and enrichment of *circREEP3* in *FKBP10* promoter was analyzed. E) *circREEP3* linearized RNA was immobilized on NC membranes, followed by probing with indicated biotin‐labeled DNA probes. F) Luciferase reporter assay using FKBP10 truncated promoters with overexpression of *circREEP3* or vector control. G,H) ChIP assay was carried out to analyze the enrichment of H3K27ac or H3K27me3 on *FKBP10* promoter. I) DNaseI accessibility assay was conducted using *circREEP3* ^+/+^ or *circREEP3* ^−/−^ LoVo cells. *n* = 3 independent samples. J) ChIP assay was conducted to detect the enrichment of RNA pol II Ser2P on *FKBP10* promoter. K) *circREEP3* ^+/+^ or *circREEP3* ^−/−^ LoVo cells were subjected to nuclear run‐on assay, followed by detection of FKBP10 transcription through qPCR analysis. *n*  =  3 independent samples. *P < 0.05 and **P < 0.01. Data were analyzed by an unpaired Student's *t*‐test and shown as means ± SD. Data are representative of at least three independent experiments.

To explore the downstream signaling, we performed RNA‐sequencing using *circREEP3* ^+/+^ and *circREEP3* ^−/−^ LoVo cells (Excel S1, Supporting Information). According to the expression fold change and value in *circREEP3* ^+/+^ LoVo cells, we selected the ten most potential candidates (**Figure** **4**A). Their expression changes were confirmed by qRT‐PCR (Figure 4B). FKBP10 was finally selected based on its high expression value and fold change. Western blot showed that *circREEP3* knockout suppressed FKBP10 protein level in LoVo cells (Figure 4C). Via chromatin isolation by RNA purification (CHIRP) assay, we found that *circREEP3* was enriched on *FKBP10* promoter (−1200 to −800 bp from the transcription start site) (Figure 4D). We also confirmed their binding through hybridization with *circREEP3* linearized RNAs (Figure 4E), which was validated via luciferase reporter assay (Figure 4F). Through ChIP assay, it was noticed that *circREEP3* knockout decreased the enrichment of active histone modification H3K27ac on *FKBP10* promoter (Figure 4G) while promoting inactive histone modification H3K27me3 enrichment (Figure 4H). To examine nucleosome density, DNase I accessibility assay was performed. *FKBP10* promoter in *circREEP3* ^−/−^ LoVo cells was more resistant to DNase I digestion (Figure 4I). Consistently, *circREEP3* knockout suppressed RNA pol II Ser2P binding to *FKBP10* promoter (Figure 4J). FKBP10 mRNA transcription was also reduced by *circREEP3* deficiency as shown by nuclear run‐on assay (Figure 4K). Thus, *circREEP3* binds to *FKBP10* promoter to initiate its transcription.

### 2.5 *circREEP3* Interacts with CHD7 to Activate FKBP10 Transcription

**Figure 5:**
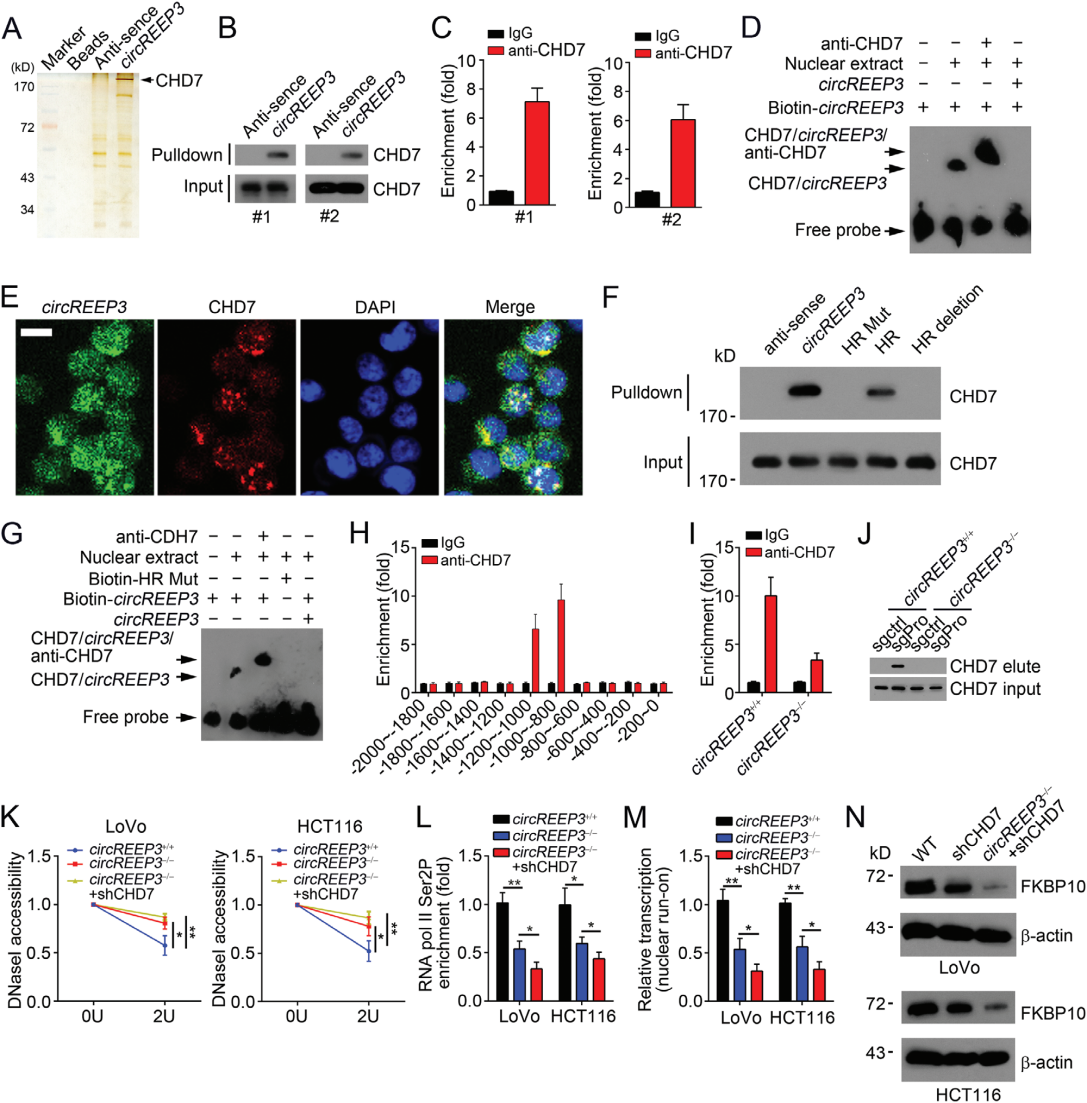
*circREEP3* interacts with CHD7 to activate FKBP10 transcription. A) CRC sample cells were lysed and incubated with biotin‐labeled and linearized *circREEP3* transcripts, anti‐sense or beads control. Precipitants were resolved by SDS‐PAGE, followed by silver staining. Indicated bands were identified via mass spectrometry. B) RNA pulldown was performed using CRC sample cell lysates and biotin‐labeled *circREEP3*. Then precipitates were detected using anti‐CHD7 by Western blotting (upper panels) and total CHD7 levels were also examined (lower panel). C) RIP (RNA Immunoprecipitation) assay was performed using anti‐CHD7 to detect the interaction between *circREEP3* and CHD7 in CRC cells. D) Electrophoretic mobility shift assay (EMSA) using biotin‐labeled *circREEP3* and nuclear extract with or without anti‐CHD7. E) *circREEP3* was colocalized with CHD7 in CRC cells by immunofluorescence staining. Scale bar, 10 µm. F) RNA pulldown assay was conducted using WT *circREEP3* and its different mutations. HR, hairpin region. The CHD7 levels in circRNA pulldown eluate (upper panel) and the input sample (lower panel) were shown. G) EMSA assay using biotin‐labeled WT *circREEP3* or indicated mutation with or without anti‐CHD7. H) ChIP assay was performed to test the enrichment of CHD7 on *FKBP10* promoter. I) ChIP assay was conducted to measure CHD7 enrichment on *FKBP10* promoter in *circREEP3* ^+/+^ or *circREEP3* ^−/−^ LoVo cells. J) Western blot of CHD7 in eluate from CAPTURE assay (CRISPR affinity purification in situ of regulatory elements) using sgRNA (small guide RNA) targeting *FKBP10* promoter. Pro, promoter. K) DNaseI accessibility assay was conducted using indicated LoVo and HCT116 cells. *n*  =  3 independent samples. L) ChIP assay was performed to measure the enrichment of RNA Pol II on *FKBP10* promoter in indicated LoVo and HCT116 cells. M) Indicated LoVo and HCT116 cells were subjected to nuclear run‐on assay, followed by detection of FKBP10 transcription through qPCR analysis. *n*  =  3 independent samples. N) Western blotting was performed to test FKBP10 expression in indicated LoVo and HCT116 cells. *P < 0.05 and **P < 0.01. Data were analyzed by an unpaired Student's *t*‐test and shown as means ± SD. Data are representative of at least three independent experiments.

To further investigate how *circREEP3* regulates FKBP10 transcription, we performed RNA pulldown assay using biotin‐labeled linearized *circREEP3* as bait, followed by silver staining and mass spectrometry. We identified that *circREEP3* interacted with CHD7 (**Figure** **5**A and Figure S2H, Supporting Information). CHD7 is a chromatin remodeler, regulating gene transcription.^[^ ^27^ ^]^ We validated the interaction between *circREEP3* and CHD7 in CRC sample cell lysates through RNA pulldown and RNA immunoprecipitation (RIP) assays (Figure 5B,C). Their direct interaction was further observed by EMSA assay (Figure 5D). Consistently, *circREEP3* was colocalized with CHD7 (Figure 5E). circRNA loops are essential for the RNA interactome.^[^ ^28^ ^]^ We analyzed the loop structure of *circREEP3* using bioinformatics tool (Figure S2I, Supporting Information). To investigate whether the loop region was important for the interaction between *circREEP3* and CHD7, we constructed hairpin region (HR) mutated *circREEP3* to abrogate the loop structure. We found that HR mutation abolished the interaction between *circREEP3* and CHD7 (Figure 5F). EMSA assay also demonstrated that the HR structure was essential for their association (Figure 5G).

To determine whether *circREEP3* recruits CHD7 to initiate FKBP10 transcription, we performed ChIP assay and found that CHD7 was enriched on the same region of *FKBP10* promoter as *circREEP3* (Figure 5H). Notably, *circREEP3* deletion impaired the enrichment of CHD7 on *FKBP10* promoter in LoVo cells (Figure 5I), which was further validated using a CAPTURE assay (CRISPR affinity purification in situ of regulatory elements) (Figure 5J).^[^ ^29^ ^]^ Besides, we found that CHD7 knockdown significantly enhanced the resistance of *FKBP10* promoter to DNase I digestion (Figure 5K). RNA pol II Ser2P enrichment on *FKBP10* promoter was further impaired by CHD7 knockdown (Figure 5L). Consistently, FKBP10 mRNA transcription was more inactive after CHD7 silencing as shown by nuclear run‐on assay (Figure 5M). Consequently, FKBP10 protein levels were decreased by CHD7 depletion (Figure 5N). Collectively, *circREEP3* recruits CHD7 to activate FKBP10 transcription.

### 2.6 *circREEP3* Promotes CRC Progression via FKBP10

**Figure 6:**
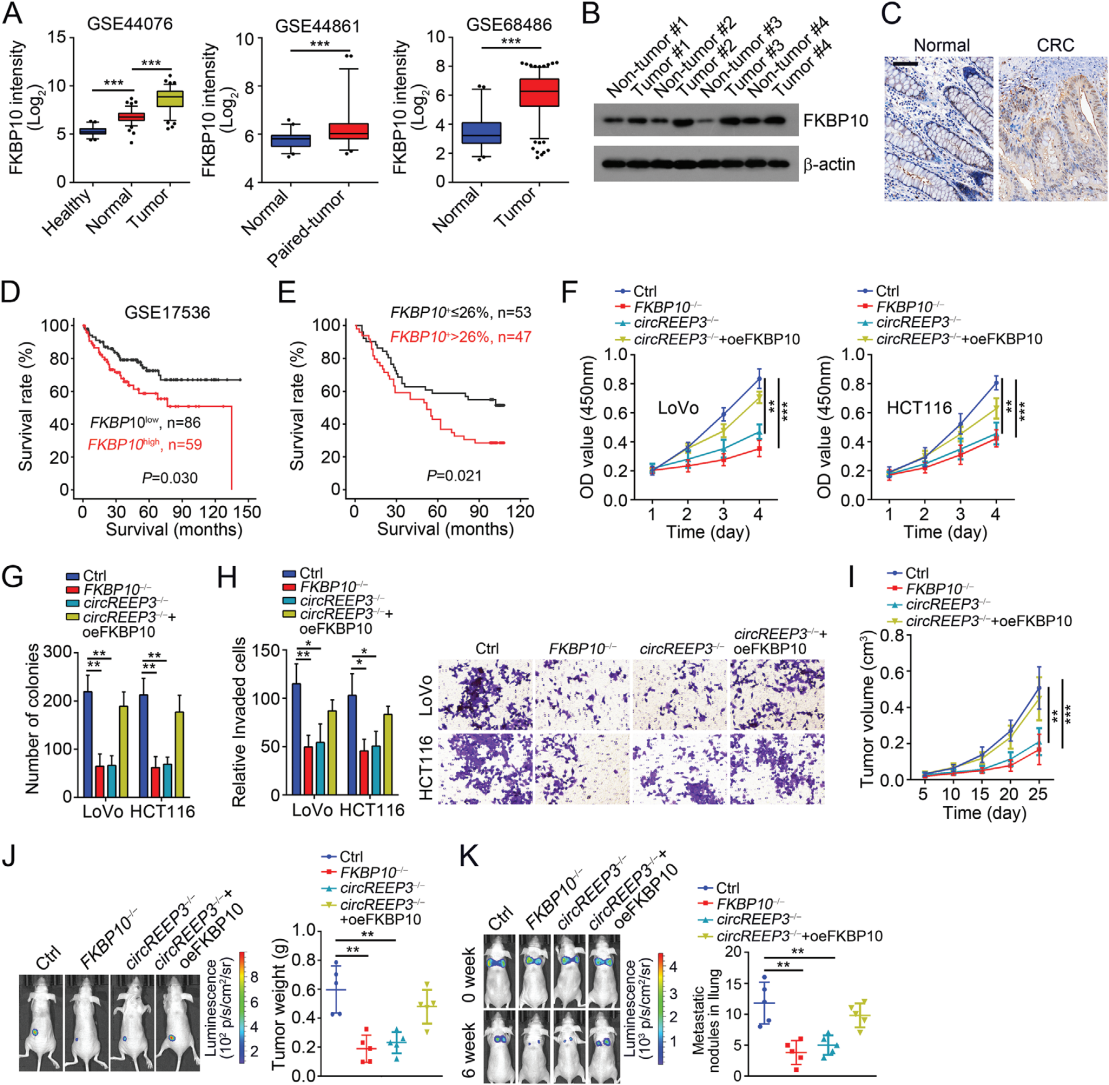
*circREEP3* promotes CRC progression via FKBP10. A) FKBP10 expression intensity was analyzed according to online data sets (GSE44076, GSE44861, and GSE68486). Healthy, healthy colon mucosa; normal, normal distant colon mucosa from tumor patients; tumor, colon cancer tissues. B) Western blotting analysis of FKBP10 expression in CRC tissues and paired adjacent normal tissues. C) FKBP10 expression was analyzed by IHC staining in paired CRC tissues and normal tissues. Scale bar, 100 µm. D) Survival rate was analyzed based on FKBP10 expression according to online data set GSE17536. E) Survival rate was analyzed by Kaplan–Meier method. CRC array tissues were divided into FKBP10 high and low subgroups based on FKBP10 expression. F,G) CCK8 and colony formation assays were conducted using indicated LoVo and HCT116 cell lines to test cell proliferation. Ctrl, control. H) Transwell assay was carried out to evaluate the invasion potential of LoVo and HCT116 cell lines. I) Indicated LoVo cells were used for xenograft assay. Tumor volumes were measured every 5 d. *n* = 5 for each group. J) Tumor weights were determined on day 25 postinjection. *n* = 5 for each group. K) Potential of lung metastasis was determined via vein tail injection of indicated LoVo cells. The number of metastatic nodules in the lung was calculated in the right panel. *n* = 5 mice each group. *P < 0.05, **P < 0.01, and ***P < 0.01. Data were analyzed by an unpaired Student's *t*‐test and shown as means ± SD. Data are representative of at least three independent experiments.

FKBP10 is a tumor driver in lung cancer.^[^ ^21^ ^]^ Whether FKBP10 affects CRC through functioning downstream *circREEP3* remains elusive. Through three independent online data sets, we found that FKBP10 expression was upregulated in CRC tissues compared to normal tissues (**Figure** **6**A). Western blot and immunohistochemistry (IHC) staining further demonstrated the overexpression of FKBP10 in CRC tissues (Figure 6B,C). We also noticed that FKBP10 expression was correlated with poor prognosis in CRC patients (Figure 6D,E), implying a potential role in tumorigenesis. Consistently, *FKBP10* knockout suppressed CRC growth and invasion in vitro while its rescue abolished the effects of *circREEP3* deficiency at least partially (Figure 6F–H). In vivo animal experiments demonstrated that CRC propagation and metastasis in lung were suppressed by FKBP10 deletion and vice versa (Figure 6I–K). These findings suggest that *circREEP3* promotes CRC growth and metastasis in a FKBP10‐dependent manner.

### 2.7 *circREEP3* Restricts Antitumor Immunity via Suppression of RIG‐1 Signaling

**Figure 7:**
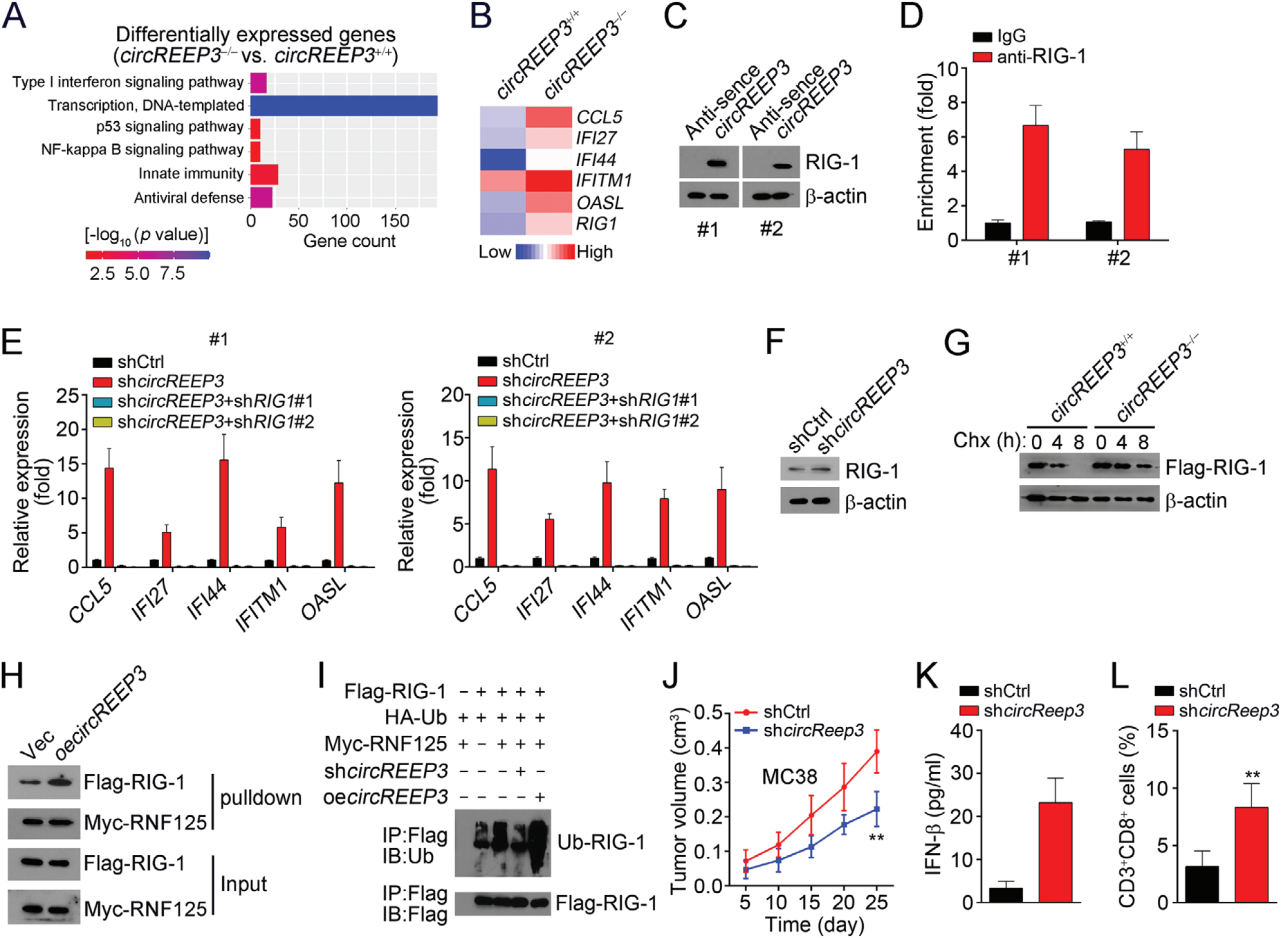
*circREEP3* restricts antitumor immunity via suppression of RIG‐1 signaling. A) GO analysis of the differentially expressed genes in *circREEP3* ^+/+^ or *circREEP3* ^−/−^ LoVo cells. B) Heatmap of RIG‐1 signaling‐related genes according to the RNA sequencing results. C) RNA pulldown was performed using CRC sample cell lysates and biotin‐labeled *circREEP3*. Then precipitates were detected using anti‐RIG‐1 by Western blotting. D) RIP assay was performed using anti‐RIG‐1 to detect the interaction between *circREEP3* and RIG‐1 in CRC cells. E) Relative expression of RIG‐1 signaling‐related genes was measured in CRC cells by qRT‐PCR. F) Western blot analysis of RIG‐1 protein levels in *circREEP3*‐silenced or control CRC cells. G) Western blot analysis of RIG‐1 protein levels in *circREEP3* ^+/+^ or *circREEP3* ^−/−^ LoVo cells transfected with Flag‐RIG‐1 vector. 36 h after transfection, the cells were treated with cycloheximide (Chx; final concentration: 50 µg mL^−1^). H) Co‐IP assay were performed using anti‐Myc and 293T lysates after transfected with Flag‐RIG‐1, Myc‐RNF125, and *circREEP3*. I) Ubiquitination signaling of RIG‐1 was measured by western blot. Indicated plasmids were transfected into 293T cells, followed by MG132 treatment. J) Tumor volumes were measured at indicated time points (*n*  =  5 mice per group). MC38 cells (5 × 10^5^) were injected subcutaneously into the right flank of C57BL/6 mice. *n* = 5 for each group. K) IFN‐*β* protein level in the serum was measured by ELISA. L) CD3^+^CD8^+^ T cells in xenograft tumors were detected by FACS. *n*  =  5 tumors per group. **P < 0.01. Data were analyzed by an unpaired Student's *t*‐test and shown as means ± SD. Data are representative of at least three independent experiments.

Interestingly, we found that *circREEP3* deletion regulated immunity‐associated pathways (**Figure** **7**A and Table S2, Supporting Information). Especially, the expression levels of several antitumor immunity‐related genes were upregulated after *circREEP3* knockout (Figure 7B). Our results from mass spectrum also indicated that RIG‐1 was a potential interactive protein of *circREEP3* (Figure S2J, Supporting Information). Through pulldown and RIP assays, the association between *circREEP3* and RIG‐1 was validated (Figure 7C,D). To explore whether *circREEP3* regulates the expression of these genes via RIG‐1, we knocked down *circREEP3* and RIG‐1 simultaneously in CRC sample cells. We found that RIG‐1 silencing abrogated *circREEP3* knockdown‐induced upregulation of CCL5, IFI27, IFI44, IFITM1, and OASL (Figure 7E), demonstrating that RIG‐1 is critical for *circREEP3*‐mediated immune signaling. Importantly, we observed that *circREEP3* knockdown increased the protein level of RIG‐1 and *circREEP3* deletion enhanced the stability of RIG‐1 (Figure 7F,G). A previous study indicates that the E3 ubiquitin ligase RNF125 promotes conjugation of ubiquitin to RIG‐I for proteasomal degradation.^[^ ^30^ ^]^ Co‐IP assay showed that *circREEP3* overexpression increased the interaction between RIG‐1 and RNF125 (Figure 7H). To determine whether *circREEP3* regulates RNF125‐mediated RIG‐1 ubiquitination, RNF125, Ub, RIG‐1, and *circREEP3* were expressed in 293T cells. We observed that the level of RIG‐1 ubiquitination was decreased by *circREEP3* knockdown while enhanced by *circREEP3* ectopic expression (Figure 7I). Therefore, *circREEP3* promotes RNF125‐dependent proteasomal degradation of RIG‐1. A recent report shows circRNA circNDUFB2 activates RIG‐1 signaling to inhibit lung cancer progression.^[^ ^31^ ^]^ To further explore whether *circREEP3* suppresses antitumor immunity in CRC, a murine CRC cell line MC38 with or without mouse *circReep3* knockdown was subcutaneously injected into C57BL/6 mice. *circReep3* knockdown significantly inhibited tumor growth (Figure 7J). The IFN‐*β* level in serum was upregulated after *circReep3* knockdown (Figure 7K). Moreover, the ratio of infiltrated CD8^+^ T cells in tumor tissues were higher after *circReep3* depletion (Figure 7L). These data suggest that *circREEP3* may also promote CRC progression by suppression of RIG‐I‐mediated antitumor immunity.

## 3 Discussion

CRC initiation and progression could be promoted by various molecules or signaling.^[^ ^32^ ^]^ However, how circRNAs are implicated in CRC progression is still poorly defined. In this study, we screened out a metastasis‐related circRNA *circREEP3*, which was highly expressed in CRC and correlated with prognosis. *circREEP3* deletion suppressed CRC cell proliferation, metastasis, and stemness. Mechanistically, *circREEP3* recruited CHD7 to initiate FKBP10 transcription in the nucleus. On the other hand, *circREEP3* enhanced the interaction between RIG‐1 and RNF125 to promote ubiquitination‐dependent degradation of RIG‐1, leading to suppression of antitumor immunity. Importantly, FKBP10 ectopic expression at least partially reversed the effects of *circREEP3* deletion on proliferation and invasion. Therefore, our findings demonstrated an essential role of *circREEP3* on CRC oncogenesis.

circRNAs are resistant to RNA exonucleases and have a long half‐life compared to other types of RNAs. Emerging research demonstrates that circRNAs are involved in the regulation of several biological processes through many mechanisms.^[^ ^33^ ^]^ Most circRNAs are found to regulate mRNA degradation by sponging miRNAs.^[^ ^34^ ^]^ Some circRNAs act as a scaffold to support the interaction among protein, DNA or RNA molecules.^[^ ^33^ ^]^ We previously found *circZbtb20* regulates mRNA stability by affecting m^6^A modification.^[^ ^16^ ^]^ We also showed *circKcnt2* could recruit chromatin remodeling complex to inhibit transcription.^[^ ^15^ ^]^ In this study, we identified that *circREEP3* recruited the chromatin remodeler CHD7 to activate FKBP10 transcription. Besides, we also showed that *circREEP3* enhanced the interaction between RIG‐1 and RNF125 and contributed to RIG‐1 degradation. Our results provide powerful evidence supporting circRNA as a scaffold.

Gene knockout is a very convincing standard to study gene function. As the development of CRISPR‐Cas9 technology, it has become very convenient to construct a knockout cell line or mice.^[^ ^35^ ^]^ Especially, knockout construction of noncoding RNAs also becomes not difficult. In the recent decade, a large number of gene knockout cell lines or mice have been obtained, which provides essential tools to understand biological problems including tumorigenesis. Recently, we investigated the roles of lncGata6 in CRC development using lncGata6 knockout mice.^[^ ^36^ ^]^ circRNA formation is dependent on the flanking complementary elements.^[^ ^7^ ^]^ Thus, deletion of the complementary elements is a strategy to construct circRNA knockout cells and mice. For example, we previously constructed circKcnt2 and circPan3 knockout mice.^[^ ^15^, ^28^ ^]^ In this study, to obtain *circREEP3* knockout cell lines, we deleted the downstream intron complementary regions using CRISPR‐Cas9 technology and validated the knockout efficiency of *circREEP3*. We found that *circREEP3* knockout remarkably inhibited CRC growth and metastasis in vitro and in vivo. Notably, *circREEP3* deletion did not alter the expression of REEP3, suggesting that *circREEP3* function is independent on its parental gene. Most circRNAs are conserved across species. We also noticed that *circREEP3* is conserved between human and mouse. Thus, using a knockout mouse model to explore the function of *circREEP3* in the future will be beneficial.

FKBP10 is a member of immunophilins that possess repeats of the peptidylprolyl isomerase (PPIase) domain.^[^ ^37^ ^]^ Interestingly, FKBP10 is located in the endoplasmic reticulum to act as protein chaperone for collagen I.^[^ ^18^ ^]^ FKBP10 has been reported to regulate osteogenesis by promoting collagen secretion.^[^ ^20^ ^]^ Recent studies have uncovered its potential functions in tumorigenesis.^[^ ^22^ ^]^ For example, FKBP10 silencing inhibits proliferation, migration, and invasion of renal cancer.^[^ ^20^ ^]^ FKBP10 contributes to glioma cell proliferation.^[^ ^38^ ^]^ Additionally, FKBP10 is a biomarker for gastric cancer progression and metastasis.^[^ ^39^ ^]^ In our study, we found that FKBP10 was upregulated in CRC tissues and may be a potential marker for prognosis. Our results demonstrated that FKBP10 knockout suppressed CRC growth and metastasis. Although FKBP10 transcription was regulated by *circREEP3*, its downstream molecular signaling remains to be determined.

Innate immune pathways such as RIG‐1 or cGAS signaling are essential protective mechanisms against cancer.^[^ ^40^ ^]^ Accumulating reports have showed that intracellular dsRNA could activate innate immune response and stimulate production of type I IFN to prevent tumorigenesis.^[^ ^41^, ^42^ ^]^ A recent work reveals that circNDUFB2 activates anti‐tumor immunity to suppress lung cancer growth.^[^ ^31^ ^]^ How circRNA regulates innate immunity in CRC is largely unknown. In our study, we found that *circREEP3* acted as a scaffold to enhance the interaction between RIG‐1 and RNF125, contributing to ubiquitination‐dependent RIG‐1 degradation. *circREEP3* knockout led to upregulated activation of RIG‐1 signaling. Using animal model, we found that *circReep3* knockdown impaired IFN‐*β* production and CD8^+^ T cell infiltration into tumor microenvironment. Thus, *circREEP3* may exert oncogenic roles through suppressing anti‐tumor immunity on the other hand, which needs more investigation using knockout mouse model in the future.

## 4 Experimental Section

### Antibodies

Anti‐H3K27ac (Cat# 8173), anti‐H3K27me3 (Cat# 9733), anti‐CHD7 (Cat# 6505), and anti‐RIG‐1 (Cat# 4200) were from Cell Signaling Technology (Danvers, USA). Anti‐REEP3 (Cat# ab241964) and anti‐FKBP10 (Cat# ab230852) were from Abcam.

### Human Samples and Cell Cultures

CRC samples were collected from Henan Provincial People's Hospital. All experiments were approved by Ethics Committee of Zhengzhou University. Fresh CRC tissue were washed two or three times and kept in DMEM/F12 medium supplemented with 1000 U mL^−1^ penicillin and 1000 U mL^−1^ streptomycin, and transferred to lab on ice quickly. The samples were washed with precooled sterile PBS (contains 100 U mL^−1^ penicillin and 100 U mL^−1^ streptomycin), and then cut into a 1 mm × 1 mm × 1 mm fragments with sterile scissors, followed by treatment with 0.25 g L^−1^ typsin/EDTA at 37 °C for 10 min, and then with 0.1 g L^−1^ type IV collagenase for 3.5 h at 37 °C. After centrifugation at 300 *g* for 5 min, CRC primary cells were collected in precipitate. Colorectal cancer cell lines were purchased from American Type Culture Collection (ATCC) and cultured using RPMI‐1640 medium (Invitrogen, Carlsbad, USA) with 10% fetal bovine serum (HyClone, USA).

### Knockout Cell Line Construction

The knockout of CRC cell lines was constructed by standard approach as previously reported.^[^ ^43^ ^]^ Generally, sgRNAs were designed and cloned into LentiCRISPRv2 (Puro, catalog no. 52961). LentiCRISPRv2, pVSVg (catalog no. 8454), and psPAX2 (catalog no. 12260) were used to generate CRISPR–Cas9 lentivirus. sgRNA sequences were listed in Table S2 (Supporting Information). All knockout cells were validated via DNA sequencing.

### Quantitative RT‐PCR Analyses

RNAs were extracted using TRIzol method. Then cDNA was synthesized with M‐MLV reverse transcriptase (Promega, Madison, USA). Gene expression was analyzed on an ABI 7300 qPCR system using specific primer pairs listed in Table S3 (Supporting Information). Relative expression levels were calculated and normalized to endogenous *ACTB*.

### Northern Blotting

Northern blotting was conducted according to a previous study.^[^ ^44^ ^]^ In brief, total RNA was subjected to formaldehyde‐denaturing agarose electrophoresis, followed by transfer to positively charged nitrocellulose (NC) membrane with 20 × SSC buffer (3.0 m NaCl and 0.3 m sodium citrate, pH 7.0). Then, the membrane was UV crosslinked and incubated with hybrid buffer for 2 h prehybridization, and then with biotin‐labeled RNA probes, which were designed to target the conjunction sequence of *circREEP3*. Biotin signals were detected with HRP‐conjugated streptavidin according to the manufacturer's instructions (Thermo Scientific).

For dot blotting, RNA was dropped onto Hybond‐N+ membrane (GE Healthcare), followed by UV crosslinking. Then RNA signal was detected using biotin‐labeled single‐stranded DNA segment. RNA was generated by in vitro transcription.

### Nuclear and Cytoplasmic Fractionation

Cytoplasmic and nuclear RNAs were obtained through using P0028 Kit (Beyotime) according to the manufacturer's instructions, followed by qRT‐PCR to detect the subcellular location of *circREEP3*.

### Colony Formation

About 500 cells per well were seeded into the six‐well plate and cultured for two weeks. Then the cells were washed using PBS, fixed in methanol for 30 min and stained using 1% crystal violet dye. Colony numbers were counted finally.

### CHIRP Assay

CHIRP assays were described previously.^[^ ^45^ ^]^ In brief, antisense DNA probes were labeled with biotin at the 3′ end. Cells were crossed with 1% formaldehyde for 10 min at 37 °C and then quenched with 0.125 m glycine buffer for 5 min. Nuclei were further lysed in nuclear lysis buffer (50 × 10^−3^ m Tris pH 7.0, 10 × 10^−3^ m EDTA, 1% SDS, add DTT, PMSF, protease inhibitor, and RNase inhibitor) on ice for 30 min and genomes were sonicated three times into 300–500 bp. Chromatins were diluted in two times volume of hybridization buffer (750 × 10^−3^ m NaCl, 1% SDS, 50 × 10^−3^ m Tris pH 7.0, 1 × 10^−3^ m EDTA, 15% formamide, add DTT, PMSF, protease inhibitor, and RNase inhibitor). Biotin‐labeled probes were added, and mixtures were rotated at 37 °C for 4 h. Streptavidin‐magnetic C1 beads were blocked with 500 ng µL^−1^ yeast total RNA and 1 mg mL^−1^ BSA for 1 h at 25 °C, and washed three times before use. Beads:biotin‐probes:RNA:chromatin were captured by magnets (Invitrogen). Finally, beads were resolved for DNA with DNA elution buffer (50 × 10^−3^ m NaHCO_3_, 1% SDS, 200 × 10^−3^ m NaCl), followed by qPCR analysis.

### Nuclear Run‐On Assay

Cells were harvested in buffer containing 150 × 10^−3^ m KCl, 10 × 10^−3^ m Tris‐HCl, 4 × 10^−3^ m MgOAc with pH 7.4, followed by centrifugation to collect cell pellets. Pellets were lysed in buffer containing 150 × 10^−3^ m KCl, 10 × 10^−3^ m Tris‐HCl, 4 × 10^−3^ m MgOAc, and 0.5% NP‐40, followed by sucrose density gradient centrifugation to prepare crude nuclei. Crude nuclei were incubated with 10 × 10^−3^ m ATP, CTP, GTP, BrUTP, and RNase inhibitor at 28 °C for 5 min. RNAs were extracted using TRIzol reagent with manufacturer's guidelines, followed by DNA digestion with DNase I. RNA transcripts were immunoprecipitated, followed by qRT‐PCR analysis.

### Luciferase Reporter Assay

FKBP10 promoter was constructed into the pGL3 vector (Promega) and transfected into 293T cells with *circREEP3* using Lipofectamine 3000 (Invitrogen). The luciferase activity was measured using the Promega dual‐luciferase assay system according to the manufacturer's instructions.

### RIP Assay

Cells were treated with 1% formaldehyde and then dissolved with RNase‐free RIPA buffer (50 × 10^−3^ m Tris‐HCl, pH 7.4, 150 × 10^−3^ m NaCl, 0.5% sodium desoxycholate, 0.1% SDS, 5 × 10^−3^ m EDTA, 2 × 10^−3^ m PMSF, 20 mg mL^−1^ aprotinin, 20 mg mL^−1^ leupeptin, 10 mg mL^−1^ pepstatin A, 150 × 10^−3^ m benzamidine, and 1% Nonidet P‐40) supplemented with protease‐inhibitor cocktail and RNase inhibitor (Roche). Supernatants were precleared with Protein A/G beads and incubated with antibodies overnight. Protein A/G beads were then added and incubated for 4 h. Total RNA was extracted from the eluent. RNA enrichment was analyzed by qPCR.

### ELISA

IFN‐*β* level in serum was detected using ELISA kit (eBioscience) according to the manufacturer's instructions.

### EMSA Assay

EMSA experiments were performed following the manufacturer's protocol with a Light Shift Chemiluminescent RNA EMSA Kit (Thermo Scientific). Briefly, nuclear extract was incubated with or without unlabeled probe for competitive reaction and anti‐CHD7 antibody for super shift at RT for 20 min in a reaction buffer. Then, biotin‐labeled probe was added into the reaction system and incubated for 20 min at RT. Samples were carried out in 4% polyacrylamide gel in 0.5× TBE buffer. After transferred on a nylon membrane (Amersham Biosciences), the labeled DNA was crosslinked by UV, probed with streptavidin‐HRP conjugate and then incubated with the detection substrate.

### RNA Pulldown

Biotin‐labeled *circREEP3* were obtained through in vitro transcription assay with biotin RNA labeling mix (Roche). CRC sample cells were lysized with RIPA buffer (150 × 10^−3^ m NaCl, 0.5% sodium deoxycholate, 0.1% SDS, 1% NP‐40, 1 × 10^−3^ m EDTA, and 50 × 10^−3^ m Tris, pH 8.0) supplemented with RNase inhibitor and protease inhibitor cocktail, and precleared with streptavidin conjugated beads for 1 h. Then biotin‐labeled *circREEP3* and cell lysates were mixed together for 3 h in 4 °C, and the biotin‐enriched components were analyzed via silver staining or western blot.

### Chromatin Immunoprecipitation

Cell lysates were crosslinked with 1% formaldehyde at 37 °C for 10 min. Then cells were washed twice with PBS, lysed with SDS lysis buffer (1% SDS, 10 × 10^−3^ m EDTA, 50 × 10^−3^ m Tris), and sonicated to make 200–500 bp DNA fragments. Lysates were precleared with Protein A Agarose/Salmon Sperm DNA (50% Slurry) and then incubated with 4 µg antibody overnight at 4 °C. Then Protein A Agarose/Salmon Sperm DNA (50% Slurry) beads were added and incubated for 4 h. After washing, DNA was eluted from beads and purified. DNA fragments were analyzed using primer pairs listed in Table S4 (Supporting Information).

### Sphere Formation

Sphere formation was performed as previously reported.^[^ ^17^ ^]^ In brief, 5000 cells were seeded into Ultra Low Attachment six‐well plates (Corning Incorporated Life Sciences, Acton, MA), and cultured in the presence of N2, B27, 20 ng mL^−1^ epidermal growth factor and 20 ng mL^−1^ basic fibroblast growth factor (Millipore). Spheres were observed and counted one week later.

### Transwell Assay

To test invasion, 2 × 10^5^ cells were seeded into 500 µL of serum‐free medium using a 24‐well Boyden chamber (BD Biosciences, NewJersey, USA) with Matrigel (BD Biosciences). FBS‐containing medium was added into the bottom chamber. After incubation for 24 h, the invaded cells in lower filters were fixed using methanol and stained with crystal violet (Sigma, MO, USA).

### Tumor Initiation Assay

For tumor initiation assay, 10, 1 × 10^2^, 1 × 10^3^, 1 × 10^4^, and 1 × 10^5^ cells were subcutaneously injected into six‐week‐old BALB/c nude mice as described before,^[^ ^46^ ^]^ followed by three months’ tumor initiation. The ratios of tumor‐free mice were calculated. *n* = 10 mice were used for each group.

### In Vivo Animal Assay

BALB/c nude mice were purchased from Vital River Laboratory (Beijing, China). All animal experiments were approved by the Ethics Committee of Zhengzhou University. For tumor growth assay, CRC cells (5 × 10^6^) were subcutaneously injected into the flank of these mice. Tumor volumes (Volume (cm^3^)  =  shorter diameter^2^  ×  longer diameter/2) were monitored every 5 d. Tumor weighs were measured on day 25 postinjection. For lung metastasis assay, 5  ×  10^5^ cells were injected intravenously into the mouse tail. Six weeks after injection, the mice were sacrificed and analyzed.

### CRISPR Affinity Purification in Situ of Regulatory Elements

CRISPR affinity purification in situ of regulatory elements (CAPTURE) assay was carried out as described before.^[^ ^29^ ^]^ Briefly, pEF1a‐BirA‐V5‐neo (addgene no. 100548), pEF1a‐FB‐dCas9‐puro (addgene no. 100547), and sgRNA targeting *FKBP10* promoter were overexpressed in CRC cells for intracellular dCas9 biotinylation and purified with Streptavidin, and the enrichment of *FKBP10* promoter binding proteins were identified through western blot.

### In Situ Hybridization

In situ hybridization was performed as previously described.^[^ ^17^ ^]^ Briefly, the tumor tissue microarray was treated sequentially by xylene, 100% ethanol, 100% ethanol, 90% ethanol, 75% ethanol for 5 min, incubated in 3% H_2_O_2_ for 15 min, followed by hybridization with *circREEP3* probes (targeting the junction sequence of *circREEP3*) under nondenatured conditions.

### Western Blot

Cell lysates were added with SDS‐loading buffer, boiled at 100 °C for 15 min, and loaded to 12% SDS‐PAGE for electrophoresis. Protein from SDS‐PAGE was transferred into the nitrate cellulose membrane, followed by incubation with primary antibodies overnight at 4 °C and HRP‐labeled secondary antibody at room temperature for 1 h. Finally, the protein levels were detected by ultrasensitive enhanced chemiluminescent (ECL) substrate.

### Microarray Assay and Transcriptome Analysis

For circRNA microarray, RNAs were isolated from CRC cells using Trizol reagent (Invitrogen) and used for Arraystar Human circRNA Array V2 (GSE196203). For transcriptome analysis, RNAs isolated from CRC cells were prepared for library construction and sequenced on DNBSEQ platform. Differentially expressed genes were identified as fold change (cutoff > 1.5, FDR < 0.05).

### Statistical Analysis

Data were analyzed by GraphPad Prism 6.0. Adobe Photoshop CC 14.0 was used for figure presentation. For statistical evaluation, an unpaired Student's *t*‐test was applied for calculating statistical probabilities in this study. For all panels, at least three independent experiments were performed with similar results, and representative experiments are shown. Data were presented as mean ± standard deviation (SD). *P*‐values ≤ 0.05 were considered significant (*P < 0.05; **P < 0.01; ***P < 0.001); *P* > 0.05, non‐significant (NS).

## Conflict of Interest

The authors declare no conflict of interest.

## Author Contribution

Z.C. and L.H. performed experiments, analyzed data, and wrote the paper; L.Z. and Z.W. analyzed data; P.Z. and B.L. initiated the study, organized, designed, and wrote the paper.

## Supporting information

Supporting Information

Supporting Information

## Data Availability

The data that support the findings of this study are available from the corresponding author upon reasonable request.
