# Supplementary material for: circREEP3 Drives Colorectal Cancer Progression via Activation of FKBP10 Transcription and Restriction of Antitumor Immunity
Source: Adv Sci (Weinh). 2022 Mar 1;9(13):2105160. doi: 10.1002/advs.202105160 (PMC9069384; doi:10.1002/advs.202105160)
Supplement: Supplementary file 1 — Supporting Information [file ADVS-9-2105160-s002.pdf]

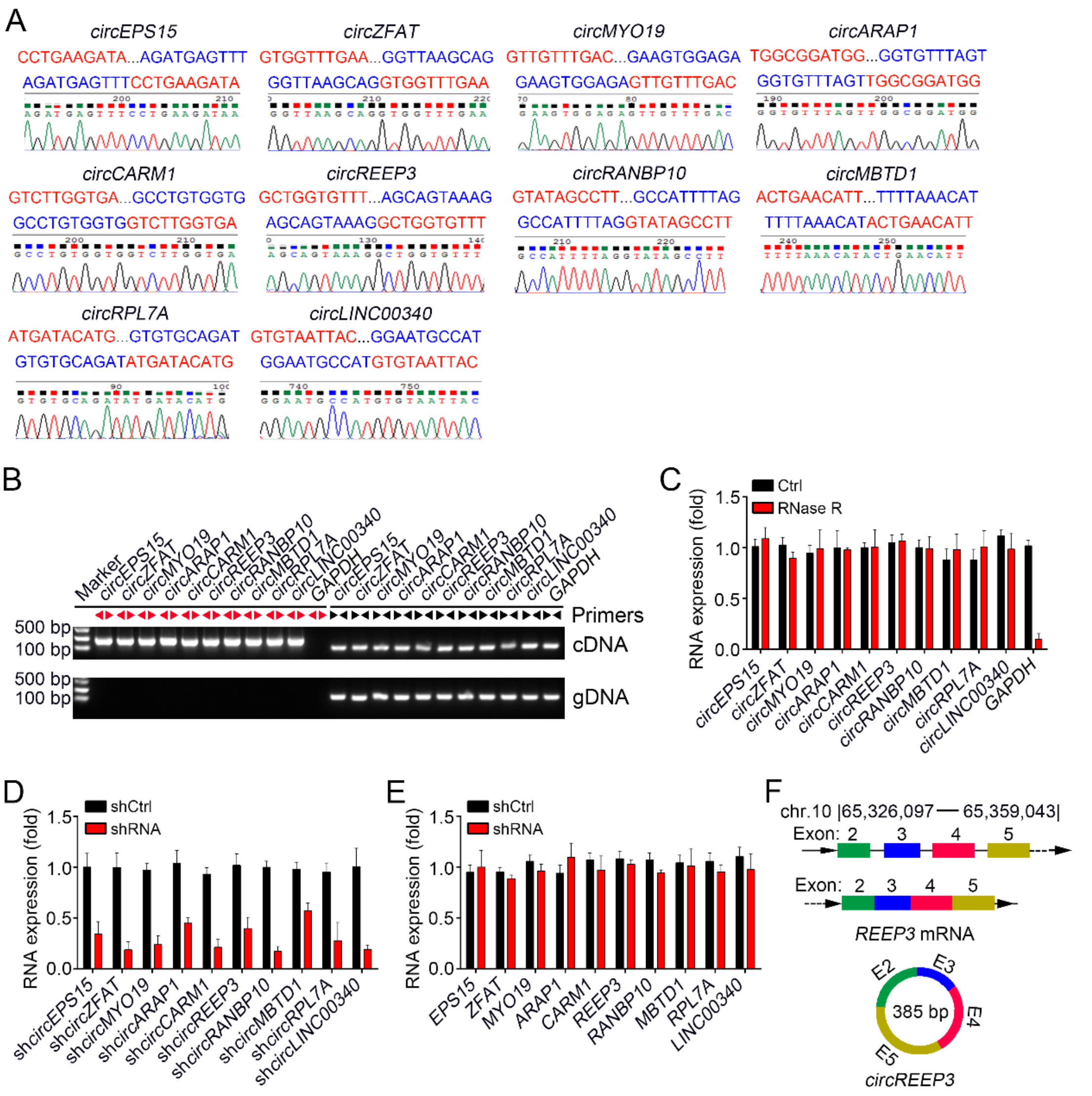

Supplementary Figure 1. Validation of upregulated circRNAs. (A) The existence of these circRNAs was confirmed by Sanger sequencing. (B) Complementary DNA (cDNA) and genomic DNA (gDNA) were used as templates to amplify circRNAs using divergent and convergent primers. Red arrowheads, divergent primers; black arrowheads, convergent primers. (C) Total RNAs were treated with or without 3 U/μg RNase R for 1 h, followed by RNA extraction and RT-PCR analysis. n = 3 independent samples. (D) CRC cells were transfected with indicated shRNAs and the knockdown efficiency was analyzed via qRT-PCR. n = 3 independent samples. (E) Relative expression levels of indicated cognate linear transcripts against respective circRNAs were measured by qRT-PCR. (F) *circREEP3* consisted of the exons from 2 to 5 of *REEP3* mRNA. Data are representative of at least three independent experiments.

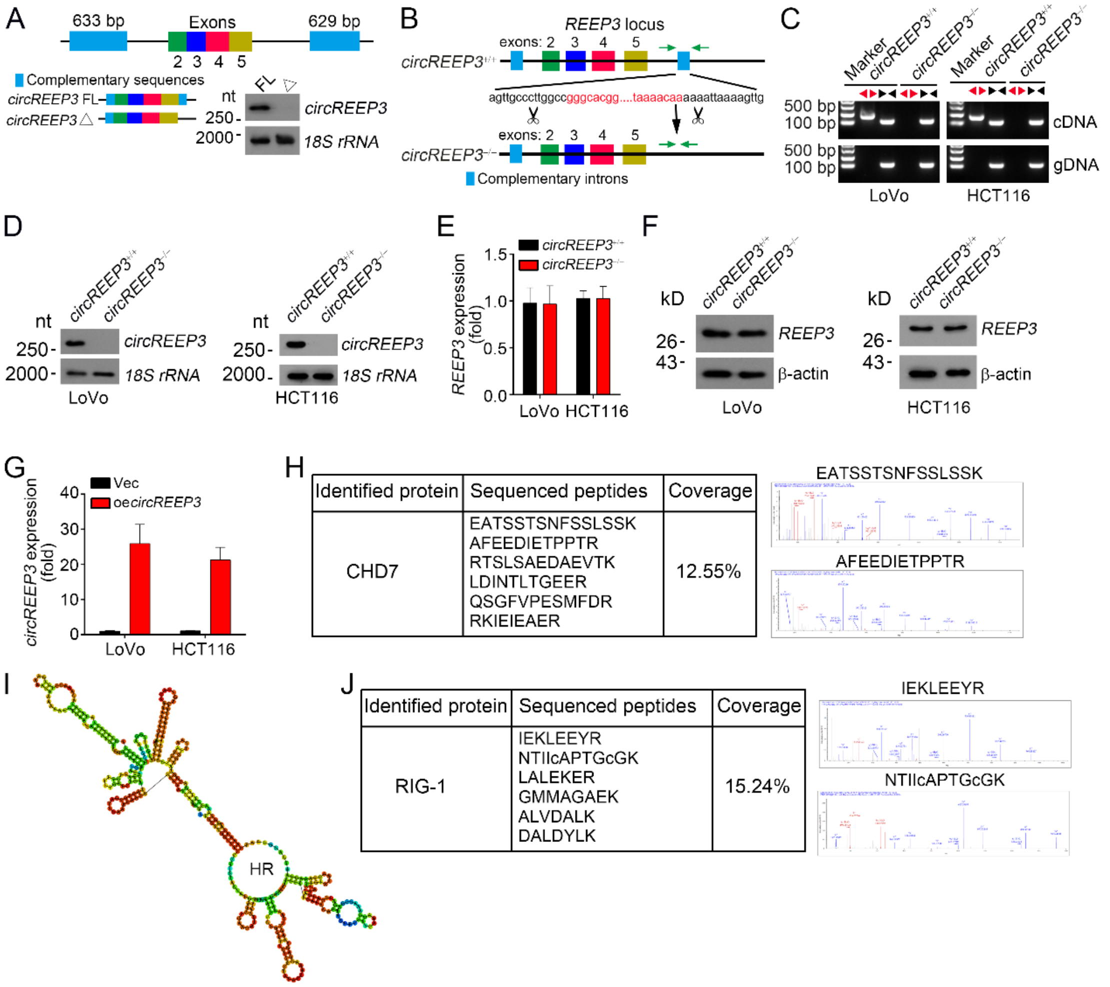

Supplementary Figure 2. Construction of *circREEP3*-deficient cell lines. (A) Diagram of exons (exon2 to exon5) and complementary introns for *circREEP3* formation. These exons and indicated complementary introns were cloned into pcDNA3 vector, followed by transfection into 293T cells for minigene assay. *circREEP3* expression was examined by Northern blotting. 18S rRNA was used as a loading control. Minigene assay validated the requirement of intronic sequences for *circREEP3* formation. (B) Diagram for construction of *circREEP3*-deficient cells. (C and D) *circREEP3* deletion was confirmed by PCR, and Northern blotting. Red arrowheads denote primers for *circREEP3* detection. Black arrowheads denote primers for liner *circREEP3* detection. n = 3 independent samples. (E and F) REEP3 levels were measured by qRT-PCR and Western blot. (G) *circREEP3* was constructed into pZW1-FCS-circRNA vector (Addgene: 73449). Then *circREEP3* upregulation was confirmed by qRT-PCR after transfection of this ectopic expression plasmid. (H) Representative peptides of CHD7 were identified by mass spectrometry. (I) Prediction of stem-loop structures of *circREEP3* through RNAfold WebServer

(http://rna.tbi.univie.ac.at/). Predictions were based on minimum free energy (MFE) and partition function. HR, hairpin region. (J) Representative peptides of RIG-1 were identified by mass spectrometry. Data are representative of at least three independent experiments.

Supplementary Table 1. Associations between *circREEP3* level and patients' clinicopathological characteristics.

Characteristics	Low (n=56)	High (n=44)	*P*-value
Age (years)			0.4536
≥60	43	37	
<60	13	7	
Gender			0.1595
Female	21	23	
Male	35	21	
Stage			0.0004
I+II	43	18	
III+IV	13	26	
Lymph node metastasis			0.0008
Yes	13	25	
No	43	19	
Diameter (cm)			0.1068
≥5	29	30	
<5	27	14	

Supplementary Table 2. GO analysis of differentially expressed genes in *circREEP3* deficient cells compared to wild-type cells.

Pathway	Gene list
Innate immunity	OAS3, OASL, CYLD, DDX58, GATA3, HERC5, ISG15, NAIP, POLR3C, SAMHD1, TBKBP1, BST2, CSF1, C1RL, C2, IFIT5, IFITM1, IRF1, IRF5 , ISG20, IL1RAP, LCN2, MYD88, PML, PRKD1, TRIM25, TRIM38, TRIM4, ZC3HAV1
Antiviral defense	OAS3, OASL, DDX58, DDIT4, HERC5, ISG15, POLR3C, SAMHD1, BST2, IFIT5, IFITM1, IRF1, IRF5, IRF9, ISG20, PLSCR1, PML, RNASEL, STAT1, STAT2, TRIM11, TRIM25, ZC3HAV1

Supplementary Table 3. sgRNAs for CRISPR/Cas9 technology

Genes	sgRNA sequences
circREEP3 (up)	5’-AAAGCTTTATACTTTAGGAGGGG-3’
circREEP3 (down)	5’-AATATGATGCTGAAATGTTGAGG-3’
FKBP10	5’-GGCACTTATGACACCTACGTCGG-3’
FKBP10 promoter	5’-TCTTAGCGCCGGGCACGAGGCGG-3’

Supplementary Table 4. Primers for PCR and qRT-PCR. (Co: Convergent primer; Di: Divergent primer)

Genes	Forward	Reverse
circEPS15 (Di)	5'-TATTGACCATGATGGAATGC-3'	5'-CAAGGATATCCACAGGTAAC-3'
circEPS15 (Co)	5'-GGCCAAATATGATGCAATAT-3'	5'-CCACAGGTAACTTAGAGTTGA-3'
circZFAT (Di)	5'-ACGGTGACTGTGGTTAAGCA-3'	5'-CTTCTGTTGCTGTCTCCCACT-3'
circZFAT (Co)	5'-AACATGGCTTGAAGGTGGTG-3'	5'-CTCTGTGATGTGGAGATACT-3'
circMYO19 (Di)	5'-ATGCAGCTGCCACAGTCATC-3'	5'-CAGGAAATGATTCAAATCCAT-3'
circMYO19 (Co)	5'-GAAACTTTGTAGAACGATAC-3'	5'-CACCATTCAGGGAGCCCTTTG-3'
circARAP1 (Di)	5'-GGAAGACCTCATTAACCACT-3	5'-CCTTGATGACTGGTGTGACG-3'
circARAP1 (Co)	5'-AGTACCGTGAGGGCAAGTAC-3'	5'-CCAGGTCTGTGGTGGTGACT-3'
circCARM1 (Di)	5'-TTCCATGGAGTGGACCTGTC-3'	5'-TGCTCGGGGAGTGACACCTC-3'
circCARM1 (Co)	5'-AGCAGGTGGACATCATCATC-3'	5'-CTGGGCTTCAGGTACTTCTT-3'
circREEP3 (Di)	5'-GCAAGGTTTAAACCTTGCAG-3'	5'-AACAATCCAGTACATCATCC-3'
circREEP3 (Co)	5'-ATCCTGCATATTATTCATACA-3'	5'-TCAATCACAGTATAGAGAGC-3'
circRANBP10 (Di)	5'-TGTGTGTGCTGCCCTCAACA-3'	5'-TGTAGTCCTCAATGTCAAAC-3'
circRANBP10 (Co)	5'-GCAGTGCTGCAGAACATGGT-3'	5'-TCTTCCTGAATCGGGGTTTC-3'
circMBTD1 (Di)	5'-TAGCAGCTCCGGTTACCTGT-3'	5'-CTTCTTCCTCACTCTCTTCG-3'
circMBTD1 (Co)	5'-AGCAGCAGCTCCAGCTCCGA-3'	5'-TCTGGGTATGTGTAGACTTG-3'
circRPL7A (Di)	5'-GCATCGTGCTCTTTGTTCTC-3'	5'-GACACTGCTACTGTACTTGT-3'
circRPL7A (Co)	5'-GTGGCCTTGAATTCAGCTTA-3'	5'-CCAAGGTGCCACGAAGTACA-3'
circLINC00340 (Di)	5'-TTGGGATGTGCTTCTGAGGT-3'	5'-ATTGTTGGCTGATGGAAAGA-3'
circLINC00340 (Co)	5'-CTGGATCTTTCCATCAGCCA-3'	5'-AAATTCCAATGATTTAACTT-3'
FKBP10	5'-GACCTGCAATGAGACCACCA-3'	5'-CCGTAGTCATGCGAGGTGAA-3'
RPL39	5'-TGCTGTCTGAAGGTCACGA-3'	5'-TGAATCCAGCCAACCAACGTG-3'
RPS29	5'- CTCGCTCTTGTCGTGTCTGT-3'	5'-AGTGCCAAGGAAGACAGCTC-3'
MICOS13	5'-GTGGATCCGAGCGACCAT-3'	5'-TTCACTTCTCATCCACGCCA-3'
PIGY	5'-CTCCAAGAAGCCGCTCAGAT-3'	5'-TCCACTTCTTCTTGCTTCTTACT-3'
H2AC12	5'-GAAGACCCGTATCATCCCGC-3'	5'-TCCAGTTTTCACAACCTCGCT-3'
SLC3A2	5'-CTGGCTCCCTCCTTTCCTTG-3'	5'-TAGGAGAAGAGTCCAGGCCC-3'
DHRS2	5'-CTGCAGACTGCCTGACAGAA-3'	5'-CTTCCTGTCTATCCCGGTGC-3'
U2AF1	5'-CCAAAACAGTGCACAGACGG-3'	5'-TCACGGCGAAACTTGACGTA-3'
CCL5	5'-TGCTGCTTTGCCTACATTGC-3'	5'-CTTGTTCAGCCGGGAGTCAT-3'
IFI27	5'-TCCTTCTTTGGGTCTGGCTG -3'	5'-TGCCTGCTCGGGTTAATTCC-3'
IFI44	5'-GTGAGGTCCAAGCTAGAGGAAG-3'	5'-TCCCTTAGATTCCCTATTTGCTCA-3'
IFITM1	5'-GCTGTCTGGGCTTCATAGCA-3'	5'-GTAGACTGTCACAGAGCCGA-3'
OASL	5'-TCGTGAAACATCGGCCAACT-3'	5'-TCGTGCCCTCTGCTGGTA-3'
REEP3	5'-GTGATGAGCCTGTGGGACAA-3'	5'-TGCGATCTTCGAAGCCCTTT-3'
ACTB	5'-CTTCGCGGGCGACGAT-3'	5'-CCACATAGGAATCCTTCTGACC-3'

Supplementary Table 5. Primers for ChIP analysis

Genes	Forward	Reverse
FKBP10 pro#1	5’-TGGCCCCCTCCTCCTATTGC-3’	5’-GGCTGGTCCACGTGGAATGG-3’
FKBP10 pro#2	5’-TTCATCTCCGCCCGGCTTTAG-3’	5’-AAGATGAGAACTCTGAGTGGA-3’
FKBP10 pro#3	5’-GTGCCCGTCACATCCCAAGTC-3’	5’-GCTCATCCGTGACCCCAGCTG-3’
FKBP10 pro#4	5’-TGAGATCAGGACACTGAGTG-3’	5’-GCAGGAGGGCGCAGCGACGGA-3’
FKBP10 pro#5	5’-TCTTAGCGCCGGGCACGAGGC-3’	5’-GAGAGGACTTAACGACACTA-3’
FKBP10 pro#6	5’-TCAGGTCCTCGGGCGGGAAG-3’	5’-CTCGGGTGGCGTGGGGGCTG-3’
FKBP10 pro#7	5’-CAGGCCCACTCGTCTGCGCG-3’	5’-TGCGCGACAGCGAGGCCTTCT-3’
FKBP10 pro#8	5’-ACCTTGAACAGCGCGTAGTG-3’	5’-TTCCAGGTGCCCTACCCGCC-3’
FKBP10 pro#9	5’-TCTGGAGGAAGGTGTAGGCC-3’	5’-CCGCGAGCTCCGGTCTTGCT-3’
FKBP10 pro#10	5’-AACACGGCCTGGAAGGGGCG-3’	5’-AGGCTTGTAGAGGAGGGCTA-3’
